# Platelet function and bleeding at different phases of childhood immune thrombocytopenia

**DOI:** 10.1038/s41598-021-88900-6

**Published:** 2021-04-30

**Authors:** Anastasia A. Ignatova, Elena V. Suntsova, Alexey V. Pshonkin, Alexey A. Martyanov, Evgeniya A. Ponomarenko, Dmitry M. Polokhov, Daria V. Fedorova, Kirill A. Voronin, Natalia N. Kotskaya, Natalia M. Trubina, Marina V. Krasilnikova, Selima Sh. Uzueva, Irina V. Serkova, Galina S. Ovsyannikova, Ksenia I. Romanova, Lili A. Hachatryan, Irina I. Kalinina, Viktor E. Matveev, Maya N. Korsantiya, Natalia S. Smetanina, Dmitry A. Evseev, Maria N. Sadovskaya, Kristina S. Antonova, Anna L. Khoreva, Pavel A. Zharkov, Anna Shcherbina, Anastasia N. Sveshnikova, Aleksey A. Maschan, Galina A. Novichkova, Mikhail A. Panteleev

**Affiliations:** 1 National Medical Research Center of Pediatric Hematology, Oncology and Immunology Named After Dmitry Rogachev, Russian Ministry of Healthcare, 1 Samory Mashela Str, Moscow, Russia 117997; 2 Shemyakin-Ovchinnikov Institute of Bioorganic Chemistry of the Russian Academy of Sciences, Moscow, Russia; 3 Center for Theoretical Problems of Physicochemical Pharmacology of the Russian Academy of Sciences, Moscow, Russia; 4 Faculty of Physics, Lomonosov Moscow State University, Moscow, Russia; 5 Institute for Biochemical Physics (IBCP), Russian Academy of Sciences (RAS), Moscow, Russia; 6 Faculty of Biology, Lomonosov Moscow State University, Moscow, Russia; 7 Department of Normal Physiology, Sechenov First Moscow State Medical University, Moscow, Russia

**Keywords:** Paediatric research, Immunological disorders, Predictive markers

## Abstract

Immune thrombocytopenia (ITP) is believed to be associated with platelet function defects. However, their mechanisms are poorly understood, in particular with regard to differences between ITP phases, patient age, and therapy. We investigated platelet function and bleeding in children with either persistent or chronic ITP, with or without romiplostim therapy. The study included 151 children with ITP, of whom 56 had disease duration less than 12 months (grouped together as acute/persistent) and 95 were chronic. Samples of 57 healthy children were used as controls, while 5 patients with leukemia, 5 with aplastic anemia, 4 with MYH9-associated thrombocytopenia, and 7 with Wiskott-Aldrich syndrome were used as non-ITP thrombocytopenia controls. Whole blood flow cytometry revealed that platelets in both acute/persistent and chronic ITP were increased in size compared with healthy donors. They were also pre-activated as assessed by PAC1, CD62p, cytosolic calcium, and procoagulant platelet levels. This pattern was not observed in other childhood thrombocytopenias. Pre-activation by CD62p was higher in the bleeding group in the chronic ITP cohort only. Romiplostim treatment decreased size and pre-activation of the patient platelets, but not calcium. Our data suggest that increased size, pre-activation, and cytosolic calcium are common for all ITP platelets, but their association with bleeding could depend on the disease phase.

## Introduction

Development of autoantibodies against platelets can lead to immune thrombocytopenia (ITP), an autoimmune disorder associated with bleeding^1,2^. Although decreased platelet count is believed to be the main cause of hemorrhage in ITP, it is a poor predictor of bleeding risk by itself. As a possible explanation of this discrepancy, there is evidence that platelet function can be impaired by antibodies as well. Several reports indicate association of platelet function in ITP with bleeding^3–6^. However, the nature and degree of this impairment are not elucidated, and there are no standardized methods of platelet function control for ITP. Furthermore, effect of therapy including new thrombopoietin mimetics, romiplostim, and eltrombopag on platelet function in ITP has been a subject of discussion as well. Differences in platelet functionality in ITP between children and adults, between acute and chronic disorder are even less clear.

We previously developed a simple comprehensive flow cytometry assay of platelet function requiring small quantities of blood and applicable in thrombocytopenia^7,8^. The limited preliminary data with this assay suggested that both children and adults with chronic ITP have abnormalities in platelet function, and that romiplostim can affect it^8–10^. However, the number and type of patients did not allow us to draw significant conclusions about these changes in children, about their clinical relevance, or about their relationship to the disease stage. Here, we investigated platelet function and calcium signal transduction in two large cohorts of children with either early-stage (acute or persistent) or chronic ITP, evaluated the effects of romiplostim and analyzed the relationship between bleeding and platelet function. In order to clearly differentiate between the effects of thrombocytopenia by itself and changes specific to immune thrombocytopenia, we additionally recruited 23 patients with thrombocytopenia caused by other hematological disorders.

## Results

### Platelet function in acute/persistent and chronic ITP, and the effects of romiplostim

**Figure 1:**
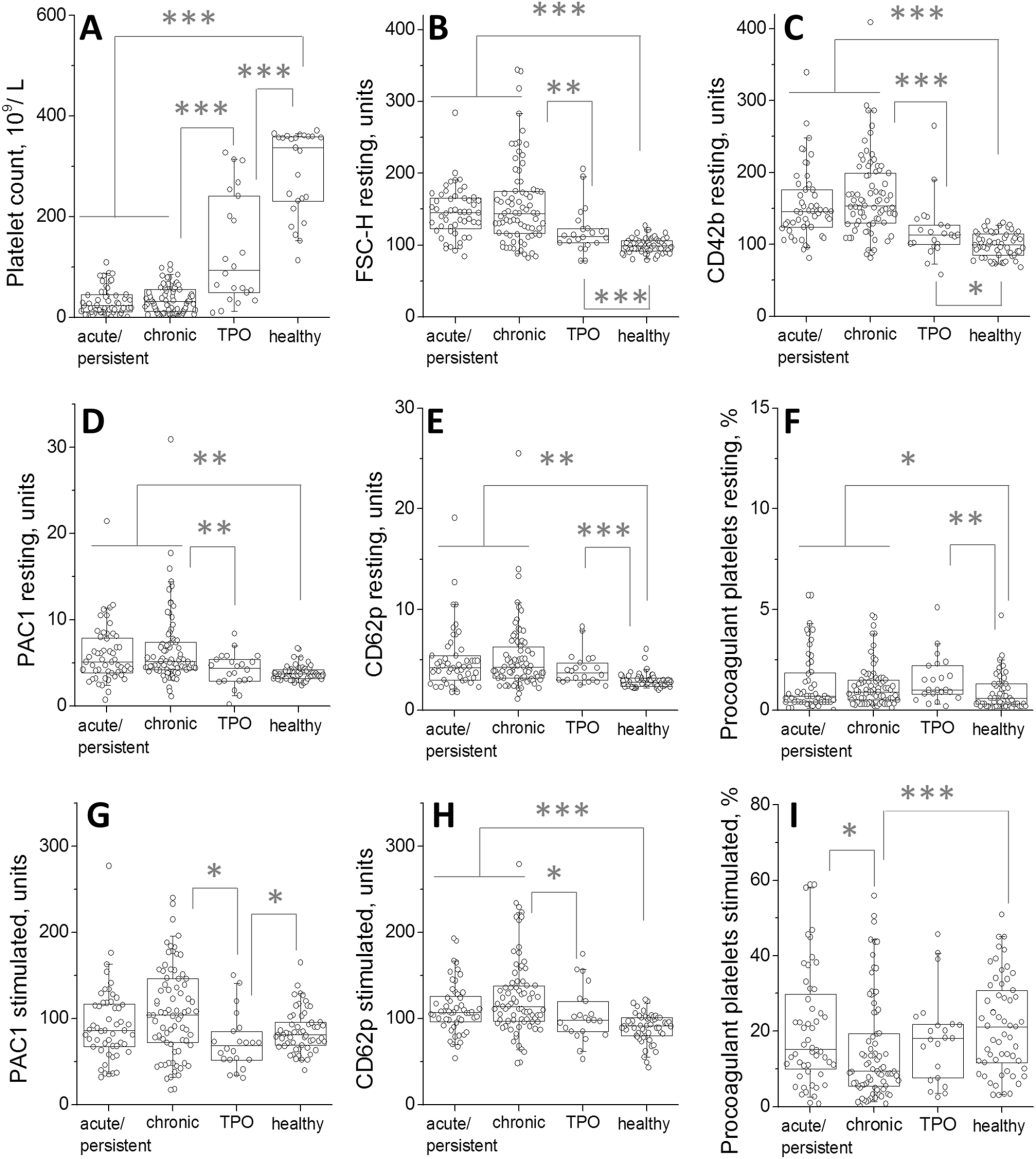
Platelet function in acute/persistent and chronic ITP, and the effects of romiplostim. The panels show platelet parameters (in resting state and upon dual stimulation by CRP + TRAP-6 mixture) for either acute/persistent ITP, chronic ITP, ITP on romiplostim (labeled as TPO), or healthy children. The data points are the circle symbols, horizontal lines are medians, boxes show 25th–75th percentiles, error bars show 5–95% intervals. Statistical significance is shown by asterisks: *, *p* < 0.05, **, *p* < 0.01; ***, *p* < 0.001.

The comparison of platelet functional activity in these three groups (Fig. 1A-I, Fig. S1) revealed a number of statistically significant shifts. Platelets from acute/persistent or chronic ITP without romiplostim treatment were significantly larger based on light scattering than those on romiplostim, which were only slightly larger than healthy controls (Fig. 1B, Fig. S1A-C). CD42b and CD61 followed the same pattern (Fig. 1C, Fig. S1D-F). Integrin activation in the resting state was increased in both untreated groups, while there was no difference between romiplostim-treated patients and healthy controls (Fig. 1D). In contrast, procoagulant platelets percentage and P-selectin were increased in all three ITP groups compared with the healthy donors (Fig. 1E, F).

Integrin activation and P-selectin expression of the non-treated ITP platelets (either acute/persistent or chronic) in response to stimulation was greater than that of romiplostim-treated group or healthy children (Fig. 1G,H). Likewise, mepacrine uptake and release in the acute/persistent or chronic ITP groups was higher than that of the treated ones (Fig. S1G-I). Interestingly, integrin activation of the romiplostim-treated group in response to stimulation was moderately lower than that of healthy children (Fig. 1H). Generation of procoagulant platelets in response to stimulation was significantly decreased in chronic ITP compared with other groups (Fig. 1I). To summarize, platelets from acute/persistent or chronic ITP patients appeared to be larger and pre-activated than normal ones, with somewhat increased response to stimulation, and differed between themselves only in procoagulant platelet generation. Those of the romiplostim-treated ones had less pre-activation and were smaller in size compared to untreated patients, although were still enlarged in size and had higher CD62p binding and levels of procoagulant platelets in the resting state compared to healthy controls.

Although the majority (60%) of patients who did not receive romiplostim were untreated at the time of study enrollment (more than a month without any treatment), the rest were receiving therapy, mainly steroids, at the time of the study or for the last month (Table 1). To analyze whether the effect of this treatment on results could be ruled out we performed the comparison of platelet functional activity between untreated patients and those receiving treatment (Fig. S2). PAC1 and CD62p binding, and procoagulant platelet levels in the resting state were noticeably lower upon treatment though the difference did not always reach statistical significance. The same was true for the parameters indicating platelet size beginning from FSC and ending with mepacrine uptake.

**Table 1:** Patient characteristics not on romiplostim.

Characteristic	Total patients	ITP duration	
		New and persistent (< 12 months)	Chronic (> 12 months)
Number	129	52 (40%)	77 (60%)
Gender			
Girls, n =	66	23	43
Boys, n =	63	29	34
Age, median (range), years	9.3 (1–18)	6.8 (1–18)	9.7 (1–18)
Platelet count, × 10^9^/L, median (range)	27 (1–109)	23 (1–109)	31 (1–105)
Medical treatment (now/last month)			
None	78/78	28/28	50/50
Any	18/33	8/16	10/17
Steroids	7/22	3/14	4/8
Not steroids	11/11	5/2	6/9
Prior therapy			
None	2	1	1
Steroids, n =	100	36	64
IVIG, n =	87	37	50
Rituximab, n =	4	0	4
IFN alpha2b, n =	28	3	25
Thrombopoietin mimetic, n =	6	0	6
Splenectomy, n =	1	0	1
Bleeding score			
0 (no bleeding),	21	12	9
1	42	15	27
2	41	21	20
3–4	25	4	21

### Platelet function and bleeding in ITP

**Figure 2:**
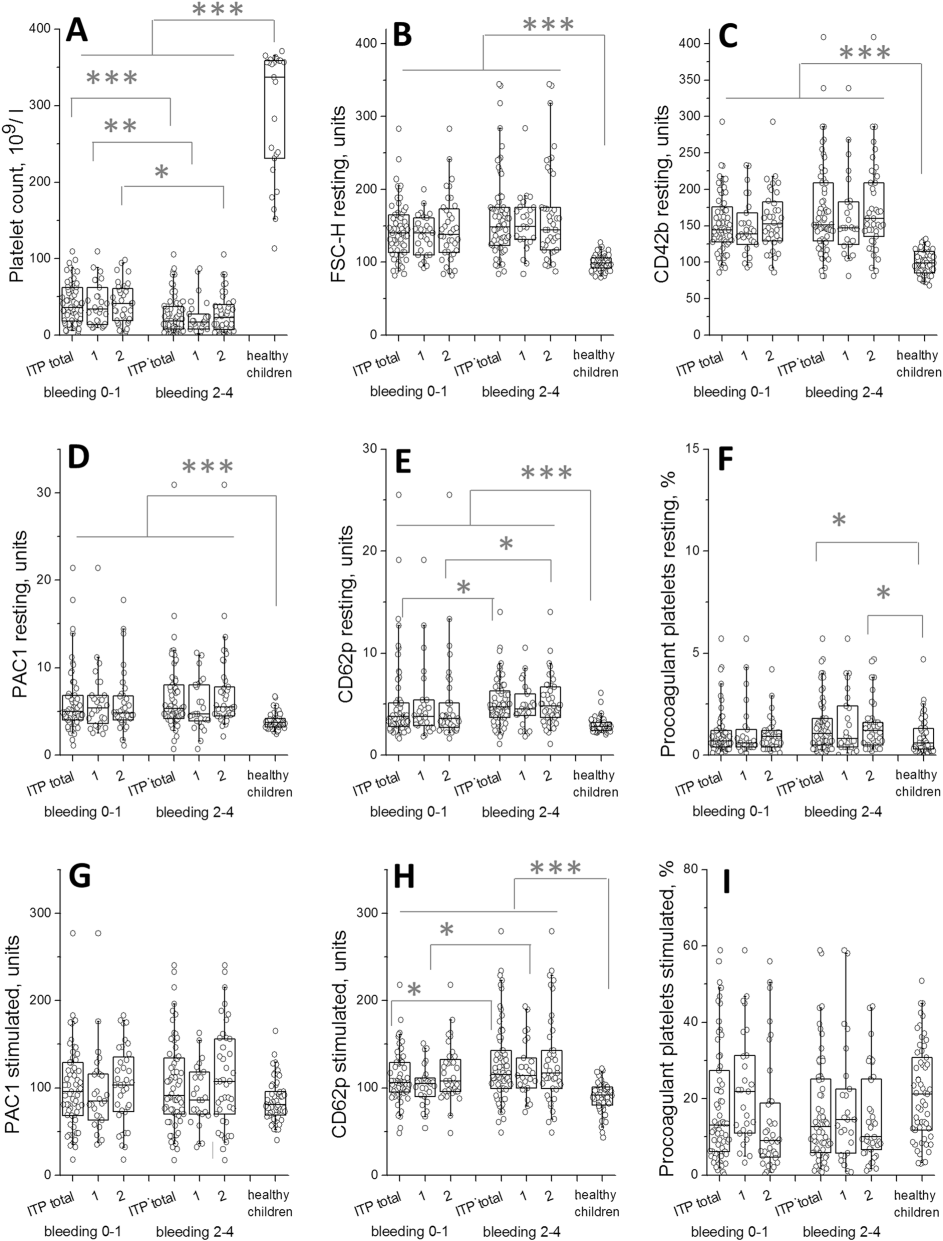
Platelet function and bleeding in ITP patients not on romiplostim. Comparison of the platelet function parameters for acute/persistent ITP (labeled as 1) and chronic ITP labeled as 2) in resting state and upon stimulation by CRP + TRAP-6 mixture. Statistics is as in Fig. 1.

In order to evaluate importance of these abnormalities for clinical manifestations, we compared platelet function of mild to severe bleeding (score 2–4) and minor or non-bleeding (score 0–1) patients (Fig. 2, Fig. S3). For both acute/persistent and chronic ITP, platelet count was significantly higher in the patients without bleeding (Fig. 2A). Resting CD62p (Fig. 2E), SSC-H and mepacrine and stimulated CD42b (Fig. S3B,D,G) were significantly higher in chronic patients with bleeding compared to those without bleeding, but were uniformly increased in the acute/persistent group (Fig. 2E, Fig. S3B,D,G). Stimulated CD62p was significantly higher in acute/persistent patients with bleeding compared to those without bleeding, but was uniformly increased in the chronic group (Fig. 2H). The other parameters behaved essentially similarly for bleeding and non-bleeding groups compared to the normal level (Fig. 2 B-D,F,G,I, Fig. S3). Comparison the bleeding between treated and non-treatment group (Fig. S4) showed no significant differences in platelet function parameters except for platelet count which was lower in acute/persistent but not chronic patients with bleeding (Fig. S4A). When the changes in platelet parameters were compared between the groups with specific bleeding scores (Fig. S5, S6), there was some indications of the differences for platelet count, as well as for CD61 after activation; however, there was no reliable dose-dependence for any of them.

### Parameter correlation and risk evaluation

To determine whether changes in the parameters and the information provided by them are independent, we investigated their correlation for different groups (Fig. S7). Size-dependent parameters as CD42b, CD61, and dense granule volume significantly correlated with FSC with correlation coefficients up to 0.79 in almost all patient and healthy children groups (Fig. S7A,B,D); for CD42b, correlation was only for the untreated patients. Resting PAC1 and CD62p also correlated with FSC in the untreated patients (Fig. S7C, S7J) suggesting that their increases could be associated with their size. However, when we normalized these data on platelet surface, the phenomenon remained (Fig. S8). In contrast, changes in procoagulant platelets were completely size-independent (Fig. S7F). FSC negatively correlated with platelet count (Fig. S7G), while resting PAC1 and procogulant platelets negatively correlated with platelet count for acute/persistent but not chronic ITP (Fig. S7H,I).

For bleeding risk stratification analysis patients were divided to two groups: with bleeding score < 2 (minor and non-bleeding group) and ≥ 2 (mild to severe bleeding group). ROC analysis of the parameters (including platelet count, FSC-H resting, PAC1 resting, CD62p resting and stimulated, procoagulant platelets resting) in relation to bleeding (Fig. S9) showed that platelet count was the best indicator of bleeding in all patient groups (acute/persistent, chronic and total ITP), with AUC values of ~ 0.65–0.72. This was also confirmed by single-variable logistic regression analysis (Supplement Table S1). Among other analyzed parameters (Supplement Table S1) stimulated CD62p was significant risk factor for acute/persistent and total ITP patient groups and FSC-H—only for total ITP patient group (*p* < 0.05).

Logistic regression model that included all the variables (platelet count, FSC-H resting, PAC1 resting, CD62p resting and stimulated, procoagulant platelets resting) revealed that platelet count remain significant predictive factor for bleeding (*p* < 0.05, Fig. S10, Supplement Table S2) in acute/persistent and total ITP, but not for chronic group. For acute/persistent ITP group it was found that PAC1 and procoagulant platelets in the resting state are significant predictive factors for bleeding (*P* < 0.05, Supplement Table S2).

### Platelet function in non-ITP thrombocytopenias

**Figure 3:**
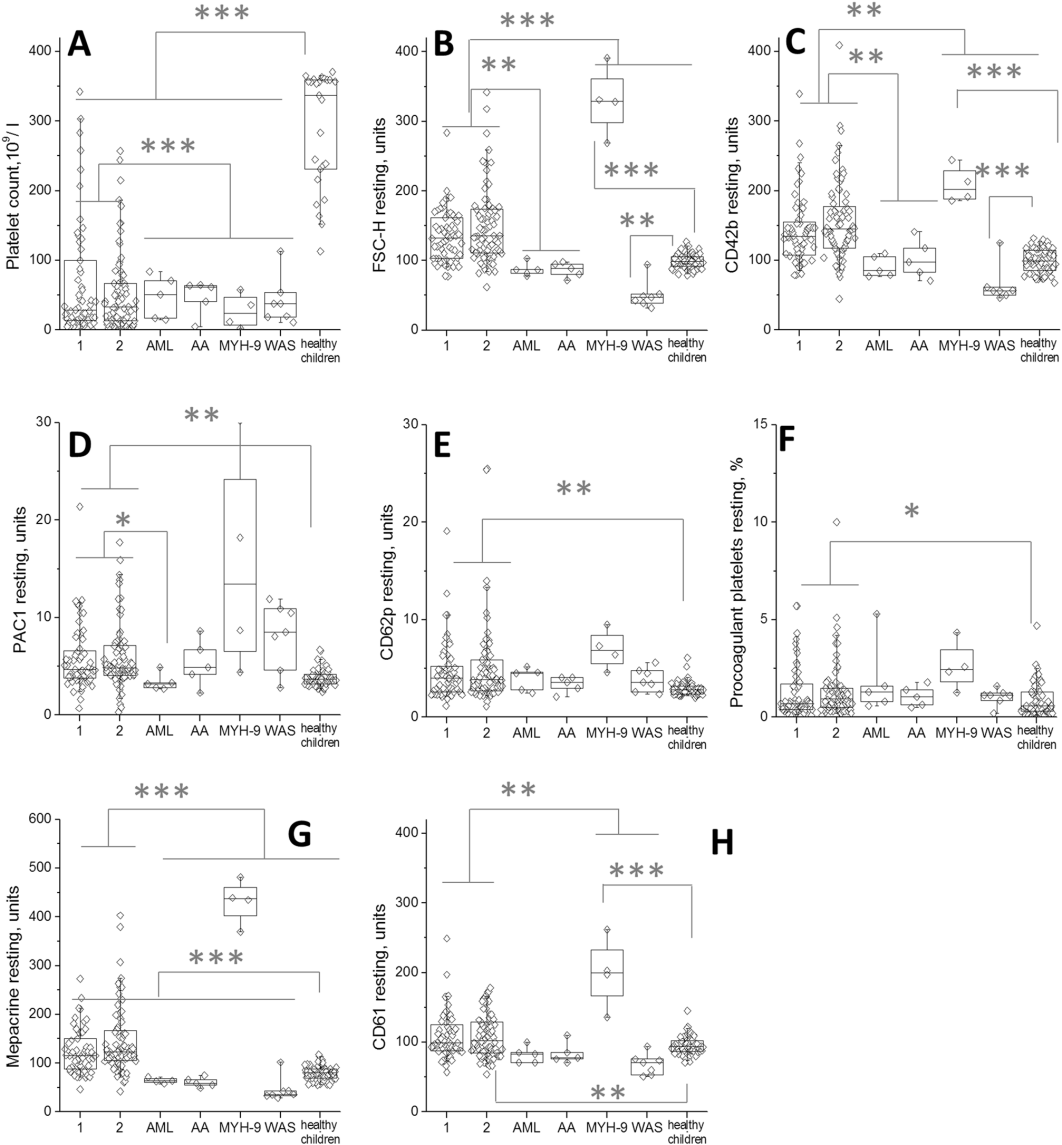
Platelet function in non-ITP thrombocytopenias. The panels show platelet function parameters for acute/persistent ITP (< 1 year, labeled as 1); chronic ITP (> 1 year, labeled as 2); leukemia (AML and JMML); aplastic anemia (AA); MYH-9 associated macrothrombocytopenia (MYH-9); Wiskott-Aldrich syndrome (WAS); and healthy donors. Statistics is as in Fig. 1.

To better discriminate between platelet function changes caused by thrombocytopenia itself and those specific for ITP, we performed experiments with samples from other thrombocytopenias (Fig. 3) that had comparable platelet counts (Fig. 3A). It can be seen from FSC that platelets in leukemia and aplastic anemia do not have any differences in size compared with healthy platelets, while WAS are smaller and MYH9 are much larger. ITP samples occupy intermediate position, being on the average larger than normal by 30–40%, but not reaching even close to the giant MYH9 platelets. Likewise, AA and leukemia(AML and JMML) were not pre-activated in PAC1, while other thrombocytopenias were. For MYH9, this could be partially explained by exceptional size increase (it was also the only thrombocytopenia except for ITP with increased CD62p), but not for WAS. With regard to the resting procoagulant platelets increase, it was completely specific to ITP. These data suggest that the "moderately increased size + preactivation in all markers" phenotype is a specific fingerprint of the ITP platelets.

### Calcium signalling in ITP

**Figure 4:**
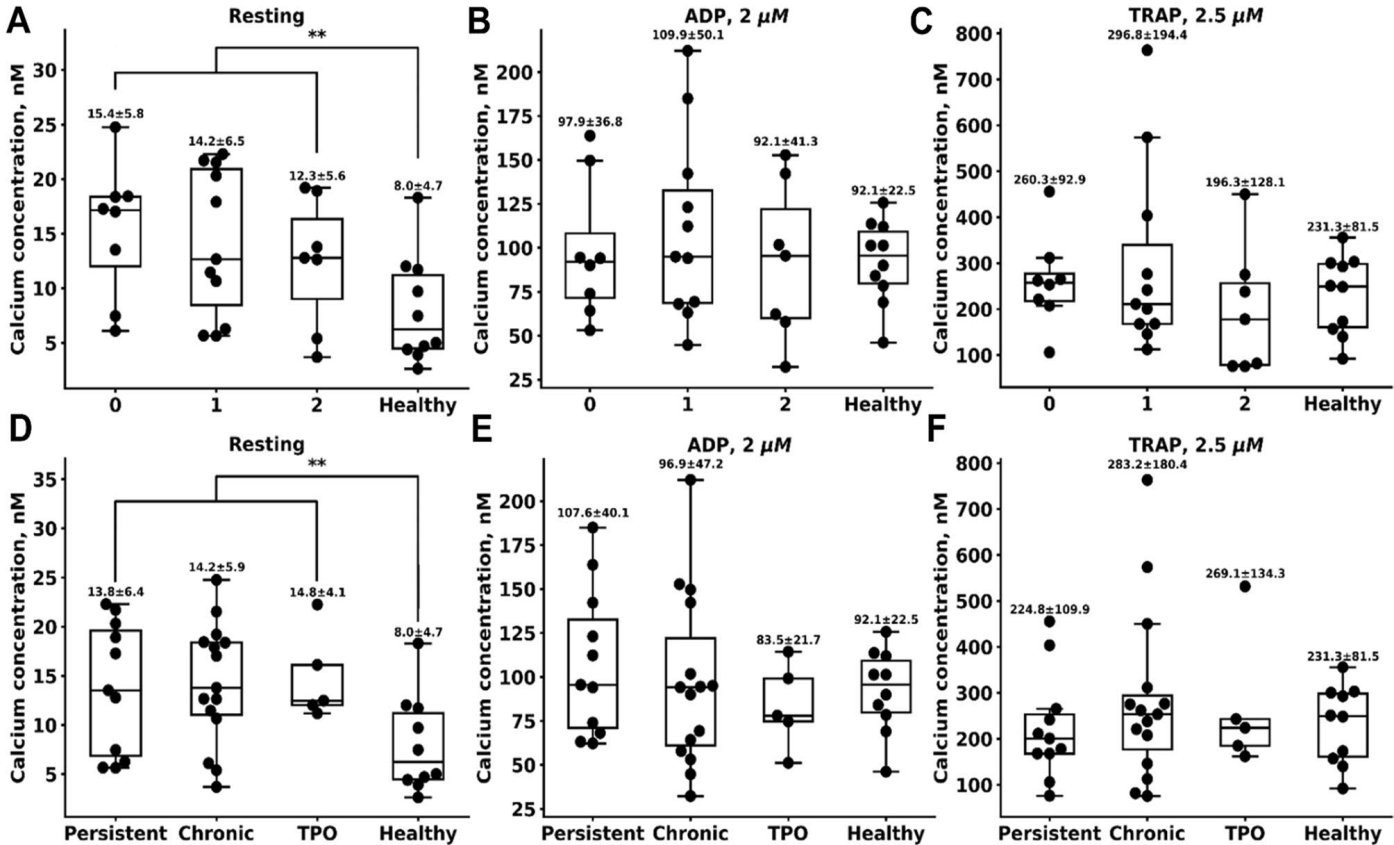
Platelet signal transduction in ITP. (**A**–**F**) The panels show platelet signalling parameters for ITP patients without bleeding (labeled as 0), mild bleeding (labeled as 1), severe bleeding (labeled as 2) and healthy children (labeled as Healthy). Cytosolic calcium concentration in resting platelets (**A**), maximal increase in calcium concentration upon activation by 2 µM of ADP (**B**) and 2.5 µM of TRAP-6 (**C**) were assessed. (**D**–**F**) The panels show platelet signalling parameters for acute/persistent ITP (< 1 year, labeled as persistent); chronic ITP (> 1 year, labeled as chronic), chronic ITP on romiplostim (labeled as TPO) and healthy children (labeled as Healthy). Cytosolic calcium concentration in resting platelets (**D**), maximal increase in calcium concentration upon activation by 2 µM of ADP (**E**) and 2.5 µM of TRAP-6 (**F**) were assessed.

In order to get insight into the mechanisms of platelet function changes in ITP, we performed analysis of cell calcium signalling in the representative patients of the above-described groups (Figs. 4 and S11). Patients of all groups had significantly higher resting cytosolic calcium level (Fig. 4A, Bleeding 0: 15.4 ± 5.8 nM, bleeding 1: 14.2 ± 6.5 nM, bleeding 2: 12.3 ± 5.6 nM; acute and persistent: 13.8 ± 6.4 nM, chronic: 14.2 ± 5.9 nM, ITP on romiplostim (TPO): 14.8 ± 4.1 nM) than healthy donors (8.0 ± 4.7 nM). Calcium responses upon activation were not altered in patients with ITP (Fig. 4B,C,E,F). Platelet size/shape (assessed by SSC) in patients with severe bleeding (7222 ± 855) was altered less than in mild (8678 ± 1315) and non-bleeding (7834 ± 1890) individuals with ITP (healthy donors: 6947 ± 1351). Neither resting calcium, nor calcium upon activation by 2 µM of ADP (except for ITP patients with mild bleeding) or 10 µM of TRAP-6 correlated with platelet FSC-H (Fig. S11A-C). On the other hand, resting calcium and calcium upon activation by 2 µM of ADP (except for ITP patients without bleeding, Fig. S11D,E, respectively), but not upon stimulation by 10 µM of TRAP-6 (Fig. S11F), correlated with resting CD62p. Finally, no correlation was observed between resting cytosolic calcium, calcium after stimulation by 2 µM of ADP and amount of procoagulant platelets after stimulation (Fig. S11G,H, correspondingly), while calcium in TRAP-6 activated platelets correlated with the amount of procoagulant platelets (Fig. S11I). Platelets of the patients with chronic ITP had increased calcium independently of romiplostim treatment (Fig. 4D). Resting calcium was increased in both chronic and acute/persistent ITP (Fig. 4D). Thus, increased basal cytosolic calcium levels in platelets of ITP patients could be associated with the pre-activation, determined by resting CD62p binding.

## Discussion

The goal of our study was to get insight into the mechanisms of platelet function and signal transduction changes in children with ITP at the early (acute/persistent, less than 12 months) and long-term (chronic, more than 12 months) phases of the disease, to study its relationship to bleeding, and to evaluate the possible effects of the romiplostim therapy. Our main results are:

1. Platelets of children in both acute/persistent and chronic ITP prior to romiplostim treatment are significantly increased in size, pre-activated, have higher cytosolic calcium level in response to TRAP-6 or ADP compared with healthy children.

2. Pre-activation determined by CD62p were higher in the bleeding group compared with non-bleeding patients, but in the chronic ITP cohort only: for acute/persistent ITP, there was no difference. In other words, pre-activation appears decreased in the chronic ITP without bleeding.

3. Romiplostim treatment significantly decreases size and pre-activation, but not the calcium level in the resting state.

It has been reported before that platelet size in ITP (which usually measured as MPV) is significantly higher than in healthy individuals or in hypoproductive thrombocytopenias^11–14^, although significantly below that in inherited macrothrombocytopenias^15,16^. Furthermore, previous reports suggested that therapy aimed at preventing hyperconsumption may decrease platelet size in ITP^17^, and its following increase could be a marker of re-lapse^18,19^. In contrast, decreased platelet size on admission in children with acute ITP was found to be a predictive marker of durable remission^20^. These observations could be mostly explained by the prevalence of large younger platelets in this hyperconsumption disorder, and increased platelet size in ITP is indeed observed side-by-side with increased immature platelet fraction^17,21^. In our previous study with chronic adult ITP, increased FSC was also significantly associated with bleeding^9^. Two studies have shown that platelet size evaluated as MPV does not correlate with bleeding score, however, even though immature platelet fraction does^3,22^. Importantly, one of them has shown that FSC does correlate with bleeding severity in a mixed cohort of ITP patients^3^. So, our results are in line with the studies reported above. The main limitation of the present study is the sample size, which should be expanded for a larger prospective trial.

In line with the FSC/MVP increase, platelet pre-activation in ITP is an accepted phenomenon, in particular with regard to the increased levels of P-selectin on resting ITP platelets^4,23–25^; few studies reported otherwise^17,26^. Although the first studies of its clinical significance suggested that this P-selectin increase was not associated with bleeding score^6^, another study found association of P-selectin with bleeding in a mixed cohort of patients^3^; we also observed association of P-selectin with bleeding in a chronic adult ITP cohort^9^. Circulating phosphatidylserine-positive platelets were also previously reported as increased in ITP ^9,27^, but not associated with bleeding; for the levels of activated integrin αIIbβ3, no significant increase in the circulation was reported.

To this pool of information, our study adds several considerations. We observed increase of almost all activation markers in the circulating platelets (P-selectin, procoagulant platelets, resting cytosolic calcium, activated integrin αIIbβ3) in acute/persistent and chronic ITP. In addition, resting CD62p was significantly lower in the group of chronic patients without bleeding; this might suggest that pre-activation is not the cause of bleeding, but rather an indicator correllating with vascular integrity issues in the chronic disease state only. Normalization of P-selectin on platelet size suggests that this pre-activation is independent of the platelet size increase. This is also supported by the correlation of the increased resting calcium (also a size-independent parameter) with increased P-selectin; this increased calcium may provide insight into the mechanisms of pre-activation. Dependence of this fact on the disease stage could explain the discrepancies in the reports described above. To summarize, the main message of our data is that pre-activation of platelets in childhood ITP is associated with calcium increase, is size-independent, is observed in both acute and chronic patient groups, but is associated with bleeding only in the chronic group.

Although it could be tempting to speculate that pre-activation could be associated with impaired platelet function, this aspect of ITP has remained much more elusive. Different studies of ITP platelet functionality by several groups including ours reported decreased adhesion^6^, impaired integrin αIIbβ3 activation^3^ (associated with bleeding), impaired aggregation and granule release^5^, normal functional responses^26,28^ or even increased ones^8,9,24^. Interpretation of these data has been complicated by the fact that platelet response could depend on the platelet count (not always accounted for in the experimental designs), on platelet size (increased in ITP), and on the activation type. In view of this discrepancy, the present study adds several important pieces of information. First, it confirms for children our previous observation in adult chronic ITP^9^: almost none of the platelet responses to potent dual-agonist stimulation is impaired in children. One important exception is procoagulant platelets formation upon activation, but we have previously shown that this is the single parameter in the assay that is platelet-concentration-dependent ^9^. On the other hand, here we developed and employed a novel assay to quantitate platelet calcium mobilization, which revealed that while childhood ITP platelets do have increased cytosolic calcium in the resting state, their calcium mobilization upon moderate stimulation with ADP or TRAP-6 is within the normal range. The calcium responses in the presence of ADP correlated with pre-activation by P-selectin, which directly indicates that pre-activation could improve the functional responses rather than impair them.

The effects of thrombopoietin receptor agonists treatment on platelet function are not clear^29^. A small-scaled study of our group^10^ followed by a larger scale study for adults^9^ indicated possibility of the pre-activation decrease on romiplostim. Here we show that, in children, P-selectin and PAC1 binding on circulating platelets but not phosphatidylserine expression are decreased upon romiplostim treatment. In line with this latter result, we show for the first time that increased cytosolic calcium level in ITP is not affected by romiplosim, which suggests a calcium-independent signalling pathway responsible for romiplostim action. Platelet activation responses to the activation (that are also in increased in untreated ITP) go down as well. A study of aggregation of platelets produced under romiplostim stimulation in ITP patients showed that these platelets have a modestly reduced aggregation response^30^. This is consistent with our finding that integrin activation upon stimulation was moderately lower in the romiplostim-treated patients compared to non-treated and even to healthy control. This also supports the overall impression that abnormalities of platelet function responses are not directly linked to bleeding. These data support the hypothesis that romiplostim might affect platelet function as well as platelet count, but the mechanisms of this action remain to be discovered. Hypothetically this may be due to platelet count normalizes on romiplostim and platelets adsorbs anti-platelet antibodies which lowers their titer. However, in our study for a small adult cohort^9^ the level of platelet-associated immunoglobulins did not change upon romiplostim treatment. Recent study^31^ shows some suggestions that murine romiplostim may affects the production of anti-platelet antibodies and reduce anti-platelet immunity, perhaps this will also be true for the human.

A limitation in our study is a lack of testing patients’ autoantibodies and their titer. A recent systematic review suggested that antiplatelet autoantibody testing is useful for ruling in adult ITP^32^, however prognostic significance of autoantibodies in childhood ITP has not yet been shown. Another limitation in our study is a lack of platelet desialylation testing. Desialilation was shown contributes to autoantibody-mediated destruction of human platelets^33,34^ and causes significant impairment of platelet function^34^. Moreover, level of platelet desialylation was correlated with response to first-line ITP treatment^35^. All of this indicates that the level of platelet desialation is an extremely important biomarker for ITP prognosis and treatment.

Taken together, our observations highlight the differences between platelet status in chronic and non-chronic childhood ITP by suggesting that platelet function changes that are distinct for ITP and are believed to be predictive of bleeding independently of platelet count, are predictive for chronic ITP only. We show that platelet pre-activation in ITP is a complex phenomenon, where not all activation markers are associated with bleeding and are not uniformly affected by romiplostim treatment. Finally, these data provide first insights into the differences in signal pathways functioning in ITP platelets, and in the effects of romiplostim on it.

## Methods

### Patients and donors

Patients aged 1 to 18 years were included in the study. Investigations were performed in accordance with the Declaration of Helsinki under a protocol approved by the CTPPCP Ethical Committee (protocol №1/2–19 from 23.12.2019), and written informed consent was obtained from all donors and patient’s parents or legal guardians. Patients were recruited at the Dmitry Rogachev National Medical Research Center of Pediatric Hematology, Oncology and Immunology (Moscow, Russia). Primary ITP was diagnosed on the basis of isolated thrombocytopenia (platelet count below 100 × 10^9^/L) with secondary thrombocytopenia excluded, according to the American Society of Hematology (ASH) Guidelines ^36^. For analysis, patients were classified into those with early stage (less than 12 months) or chronic (more than 12 months) disease. Current bleeding (at the day of blood collection) was graded using the Buchanan bleeding score ^37^. The control groups were composed of healthy children and of patients with thrombocytopenias of different origins. The underlying disorders were diagnosed on the basis of commonly accepted criteria, and confirmed genetically for the inherited disorders.

The study included 129 ITP patients without romiplostim treatment (Table 1): 78 girls and 73 boys aged 1–18 years (median 9 years). The majority of them (63%) had chronic ITP. The average platelet count was 33 × 10^9^/L. The majority of patients (75%) had clinically significant bleeding manifestations (higher number indicates more severe bleeding): 1st degree, 46; 2nd degree, 41; 3rd degree, 23; 4th degree, 2. Almost all patients (127) had a history of 1 to 4 lines of therapy, and 2 patients did not receive specific therapy before. The group on romiplostim treatment (Table 2) included 22 patients, of whom 18 had chronic ITP and 4 had acute/persistent ITP. Of them, 15 patients received romiplostim as monotherapy and 7 received it in combination with steroids and/or IVIG. The duration of romiplostim treatment ranged from 2 to 180 weeks (median 8 weeks). All patients on romiplostim had 2 to 4 lines of prior therapy, one had splenectomy, and one had previous experience of a thrombopoietin mimetic (eltrombopag). Among them, 13 had stable response to romiplostim treatment.

**Table 2:** Patient characteristics on romiplostim.

Characteristic	
Number	22
Gender	
Girls, n =	12
Boys, n =	10
Age, median (range)	10 (2–16)
Platelet count, × 10^9^/L, median (range)	94 (9–327)
Prior therapy	
Steroids, n =	22
IVIG, n =	20
Rituximab, n =	3
IFN alpha2b, n =	6
Thrombopoietin mimetic, n =	1
Splenectomy, n =	1

The control patients with non-ITP thrombocytopenias (Table S3) were: acute myeloid leukemia, 5 (including 1 juvenile myelomonocytic leukemia); aplastic anemia, 5 (including 4 patients with acquired and 1 patient with constitutional disease); MYH-9 associated macrothrombocytopenia, 4; and Wiskott-Aldrich syndrome, 7. Healthy children (n = 57, aged 1 to 18 years) were enrolled as a control. All of them did not have thrombocytopenia or bleeding of any kind. They were not given anti-platelet, anti-inflammatory drugs or antibiotics either.

### Materials

Annexin V-Alexa647 and antibodies against P-selectin (CD62p-Alexa647), glycoprotein I (CD42b-PE), integrin αIIβ3 (CD61-PE) and its activation marker (PAC1-FITC) were from Sony Biotechnology (San Jose, CA, USA). Cysteine-containing version of cross-linked collagen-related peptide (CRP) was custom-synthesized and purified by VCPBIO (Shenzhen, China) and then cross-linked. All other reagents were from Sigma-Aldrich (St Louis, MO, USA).

### Flow cytometry evaluation of platelet function

Platelet function was analyzed as in ^8,10,25^ with minor modifications. Blood was collected by venipuncture into 3-ml vacuum citrate tubes. Whole blood samples were diluted 1:20 with buffer A (150 mM NaCl, 2.7 mM KCl, 1 mM MgCl_2_, 0.4 mM NaH_2_PO_4_, 20 mM HEPES, 5 mM glucose, 0.5% bovine serum albumin, pH 7.4^38,39^). Platelets were either left intact or loaded with mepacrine (10 µM) for 30 min at 37 °C. Subsequently, they were either left unstimulated or stimulated with CRP at 20 µg/µL and TRAP-6 at 12.5 µM for 10 min in the presence of 2.5 mM calcium chloride. Both resting and activated samples were incubated with antibodies against CD61, CD42b, CD62p, as well as PAC1 and annexin V for 10 min. Subsequently, they were diluted tenfold with buffer A containing 2.5 mM calcium, and analyzed using Novocyte (Acea Bioscience, San Diego, CA, USA) flow cytometer.

### A dynamic assay of platelet calcium mobilization

For platelet calcium measurements blood was collected by venipuncture in hirudin vacuum tubes. Whole blood was incubated at 37 °C in the presence of 2 µM of Fura-Red and 0.1 U/mL of apyrase for 35 min. Blood plasma was collected from above the settled red blood cells and resuspended in Tyrode’s (150 mM NaCl, 2.7 mM KCl, 1 mM MgCl_2_, 2 mM CaCl_2_, 0.4 mM NaH_2_PO_4_, 0.4 mM Na_2_CO_3_, 5 mM HEPES, 5 mM glucose, 0.5% BSA, pH 7.35) buffer to the final concentration of 10^3^ µL^-1^. Diluted platelets rested for 30 min at 37 °C. Samples were then analyzed using BD FACS Canto II flow cytometer. Ratio of Fura-Red direct (405 nm excitation) to Fura-Red inverted (488 nm excitation) was recalculated to the cytosolic calcium concentration using Grynkiewicz formula after calibration with 1 µM of ionomycin and 10 mM of EGTA^40^. Additional calibration was performed upon calculation of the free calcium concentration in the presence of 10 mM of EGTA based on^41^. Typical calcium response on activation are given in Fig.S12. Calcium mobilization was calculated by subtraction of resting calcium concentration from maximal calcium concentration.

### Statistics

The results were analyzed using Origin 8.0 software (OriginLabCorp., Northampton, MA, USA). Comparison of the parameters between patients and healthy donor groups was performed using Mann–Whitney non-parametric test. The significance level was set as 95%. Spearman’s correlation coefficient was used to assess the parameter’s correlation. To determine the significance of the correlations, the 2-tailed test of significance was used. To compare the bleeding risk prediction by different parameters the receiver operating characteristic (ROC) curve and the area under it were utilized. To obtain odds ratio (OR) logistic regression analysis was performed in SPSS Statistics software version 26.0.0.0 (IBM, New York, USA).

### Data sharing statement

For original data, please contact corresponding author.

## Supplementary Information

Supplementary Figures.

Supplementary Tables.

## Abbreviations

AML: Acute myeloid leukemia
ITP: Immune thrombocytopenia
OR: Odds ratio
ROC curve: Receiver operating characteristic curve
JMML: Juvenile myelomonocytic leukemia
