# Supplementary material for: Platelet function and bleeding at different phases of childhood immune thrombocytopenia
Source: Sci Rep. 2021 Apr 30;11:9401. doi: 10.1038/s41598-021-88900-6 (PMC8087794; doi:10.1038/s41598-021-88900-6)
Supplement: Supplementary file 1 — Supplementary Figures. [file 41598_2021_88900_MOESM1_ESM.pdf]

Platelet function and bleeding at different phases of childhood immune thrombocytopenia

Anastasia A. Ignatova[1,2,3*], Elena V. Suntsova [1*], Alexey V. Pshonkin [1], Alexey A. Martyanov[1,3,4,5], Evgeniya A. Ponomarenko[1,6], Dmitry M. Polokhov[1], Daria V. Fedorova[1], Kirill A. Voronin[1], Natalia N. Kotskaya[1], Natalia M. Trubina[1], Marina V. Krasilnikova[1], Selima Sh. Uzueva[1], Irina V. Serkova[1], Galina S. Ovsyannikova[1], Ksenia I. Romanova[1], Lili A. Hachatryan[1], Irina I. Kalinina[1], Viktor E. Matveev[1], Maya N. Korsantiya[1], Natalia S. Smetanina[1], Dmitry A. Evseev[1], Maria N. Sadovskaya[1], Kristina S. Antonova[1], Anna L. Khoreva[1], Pavel A. Zharkov[1], Anna Shcherbina[1], Anastasia N. Sveshnikova[1,3,4,7], Aleksey A. Maschan[1], Galina A. Novichkova[1], Mikhail A. Panteleev[1,3,4,8]

[1] National Medical Research Center of Pediatric Hematology Oncology and Immunology named after Dmitry Rogachev, Moscow, Russia
[2] Shemyakin-Ovchinnikov Institute of Bioorganic Chemistry of the Russian Academy of Sciences, Moscow, Russia
[3] Center for Theoretical Problems of Physicochemical Pharmacology of the Russian Academy of Sciences, Moscow, Russia
[4] Faculty of Physics Lomonosov Moscow State University, Moscow, Russia
[5] Institute for Biochemical Physics (IBCP), Russian Academy of Sciences (RAS), Moscow, Russia
[6] Faculty of Biology Lomonosov Moscow State University, Moscow, Russia
[7] Department of Normal Physiology Sechenov First Moscow State Medical University, Moscow, Russia
[8] Faculty of Biological and Medical Physics Moscow Institute of Physics and Technology, Dolgoprudny, Russia
* Equal contribution

Supplement figures

Supplement figure S1

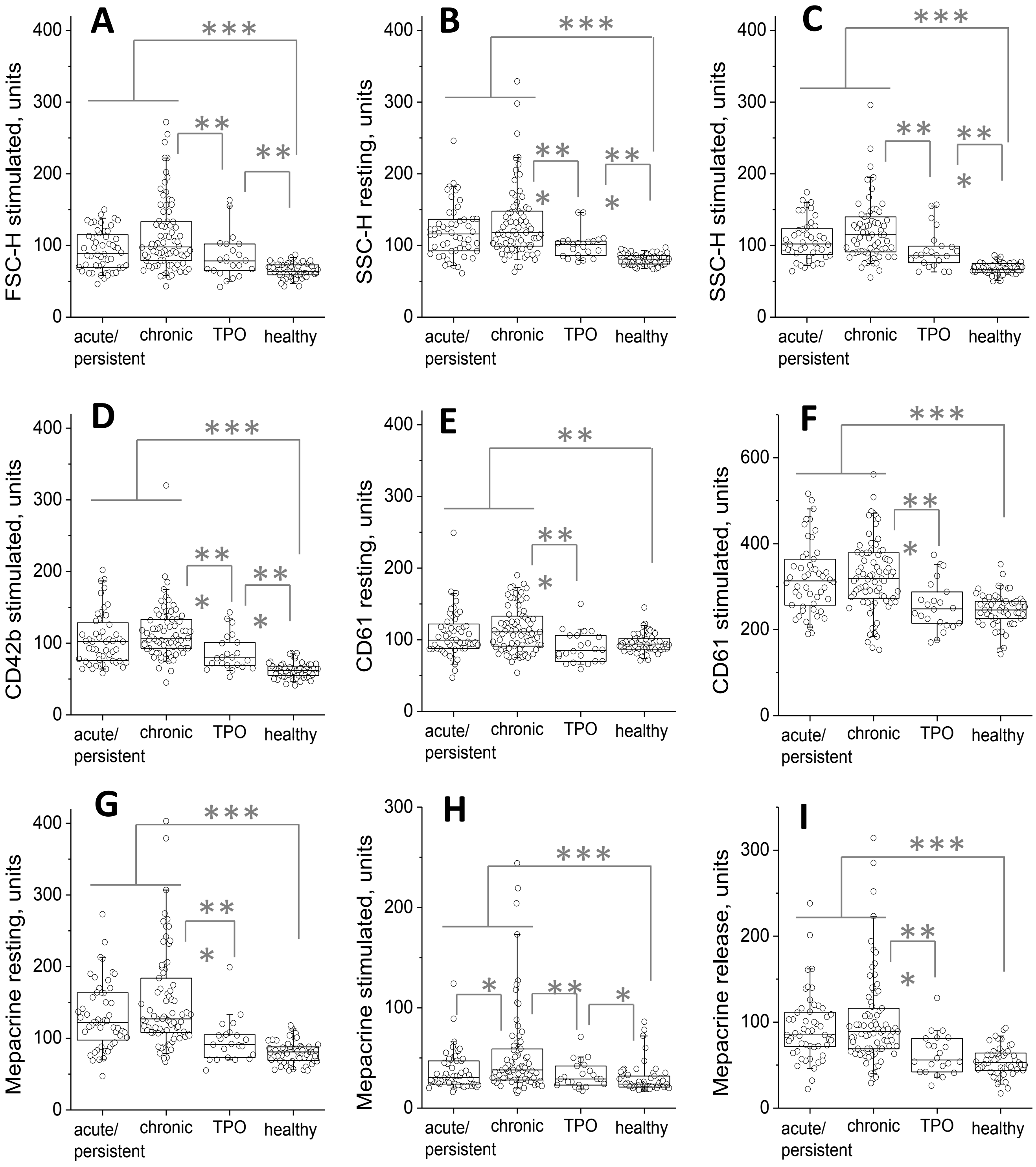

Figure S1. Platelet function in acute/persistent and chronic ITP, and the effects of romiplostim. All conditions and notation are as in Fig. 1.

Supplement Figure S2

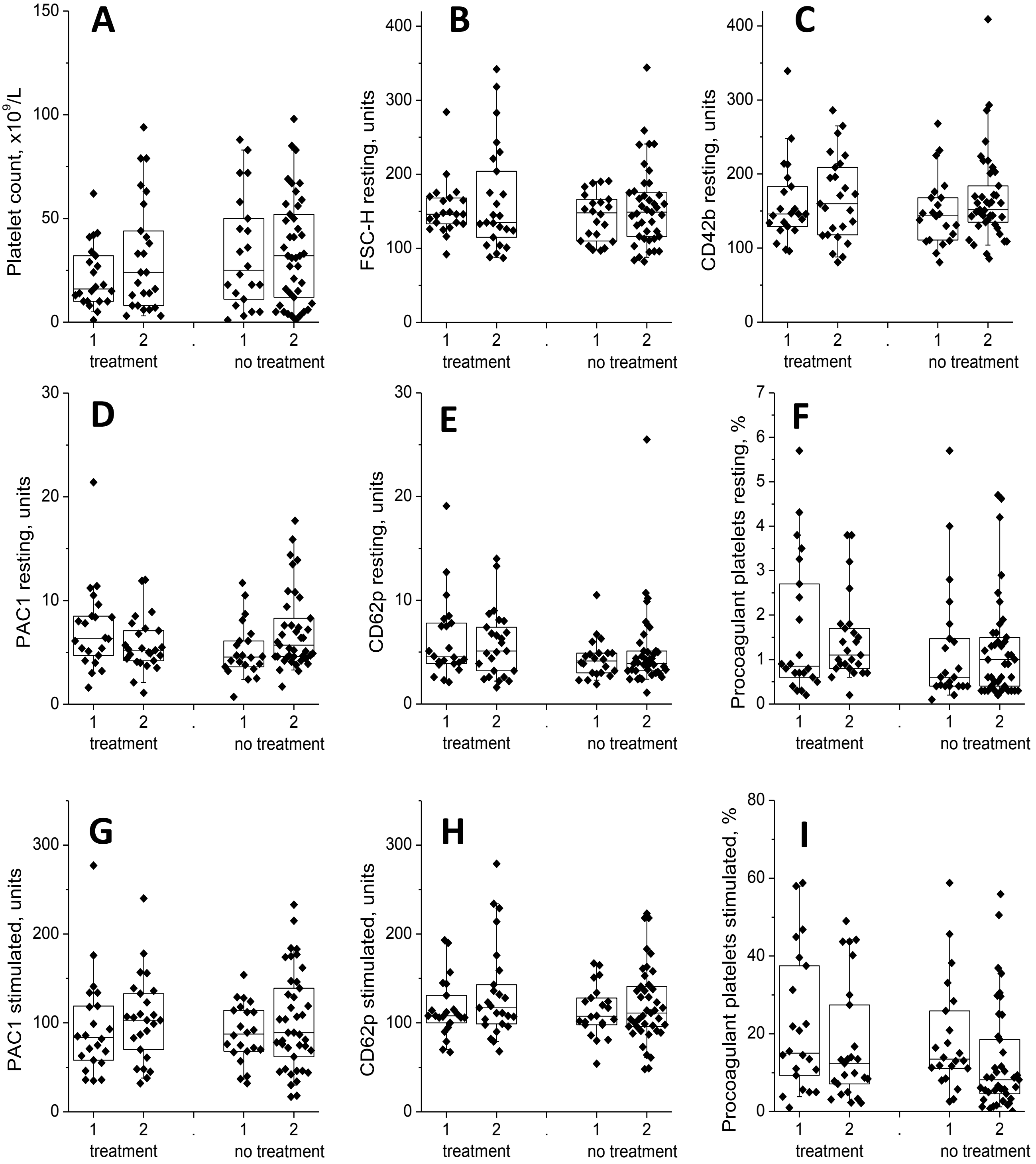

Figure S2. Comparisons for platelet function parameters between *acute/persistent (1) and chronic (2)*untreated *ITP* patients and those receiving treatment (mainly steroids). (Pairwise comparisons were made by Dunn-Bonferroni post hoc method following a significant Kruskal-Wallis test).

Supplement figure S3

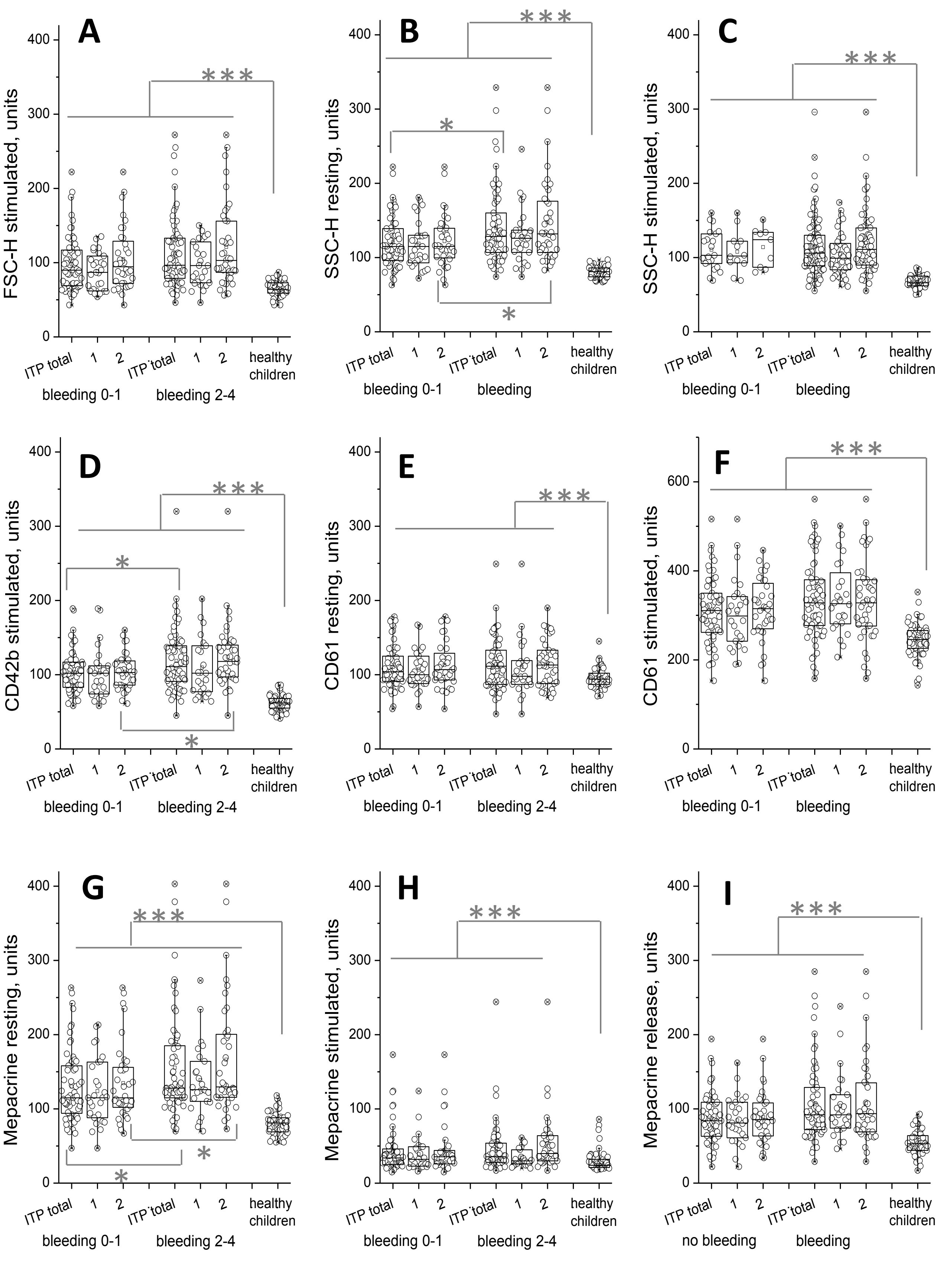

Figure S3. Platelet function and bleeding in non-treated ITP. All conditions and notation are as in Fig. 2.

Supplement Figure S4

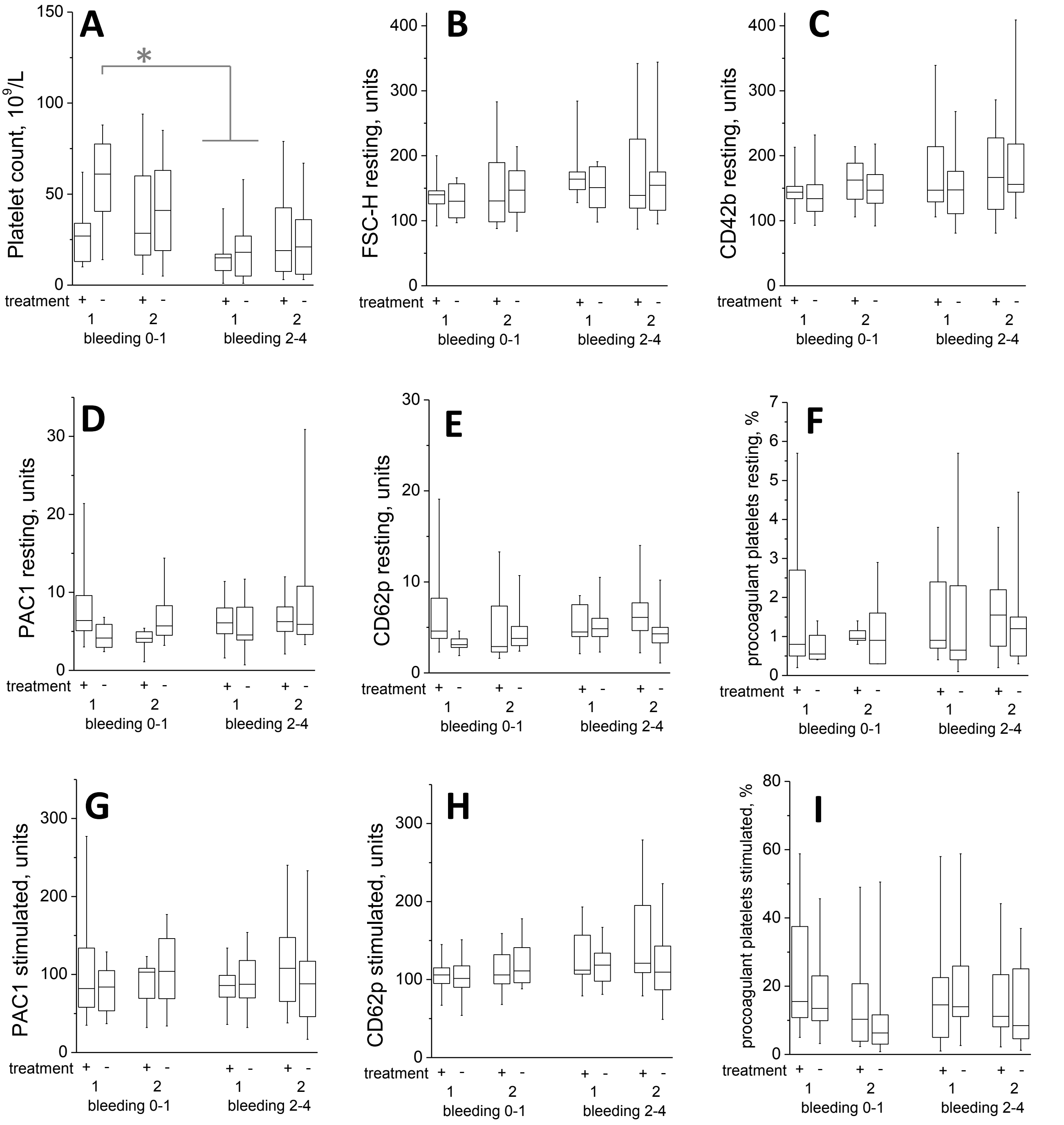

Figure S4. Effect of treatment (mainly steroids) on platelet function and bleeding degree in acute/persistent (1) and chronic (2) ITP patients non-treated with romiplostim. Horizontal lines are medians, boxes show 25th-75th percentiles, error bars show 5-95% intervals. Stimulation with CRP+TRAP6 mixture was used. Statistical significance is shown by asterisks: *, $p<0.05$ (Pairwise comparisons were made by Dunn-Bonferroni post hoc method following a significant Kruskal-Wallis test).

Supplement Figure S5

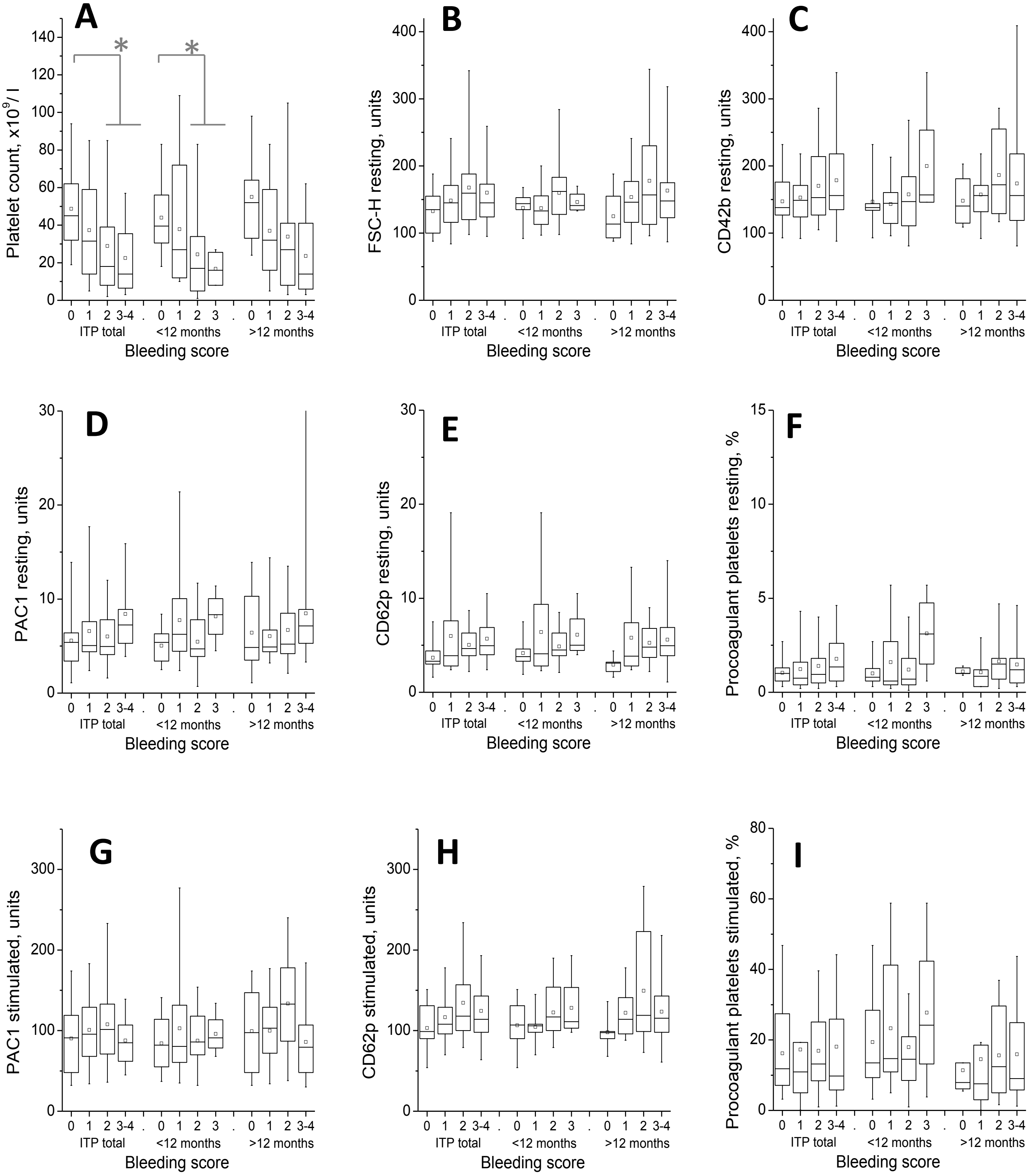

Figure S5. Platelet function and bleeding degree in ITP non-treated with romiplostim. Horizontal lines are medians, boxes show 25th-75th percentiles, error bars show 5-95% intervals. Statistical significance is shown by asterisks: *, $p<0.05$ (Pairwise comparisons were made by Dunn-Bonferroni post hoc method following a significant Kruskal-Wallis test).

Supplement Figure S6

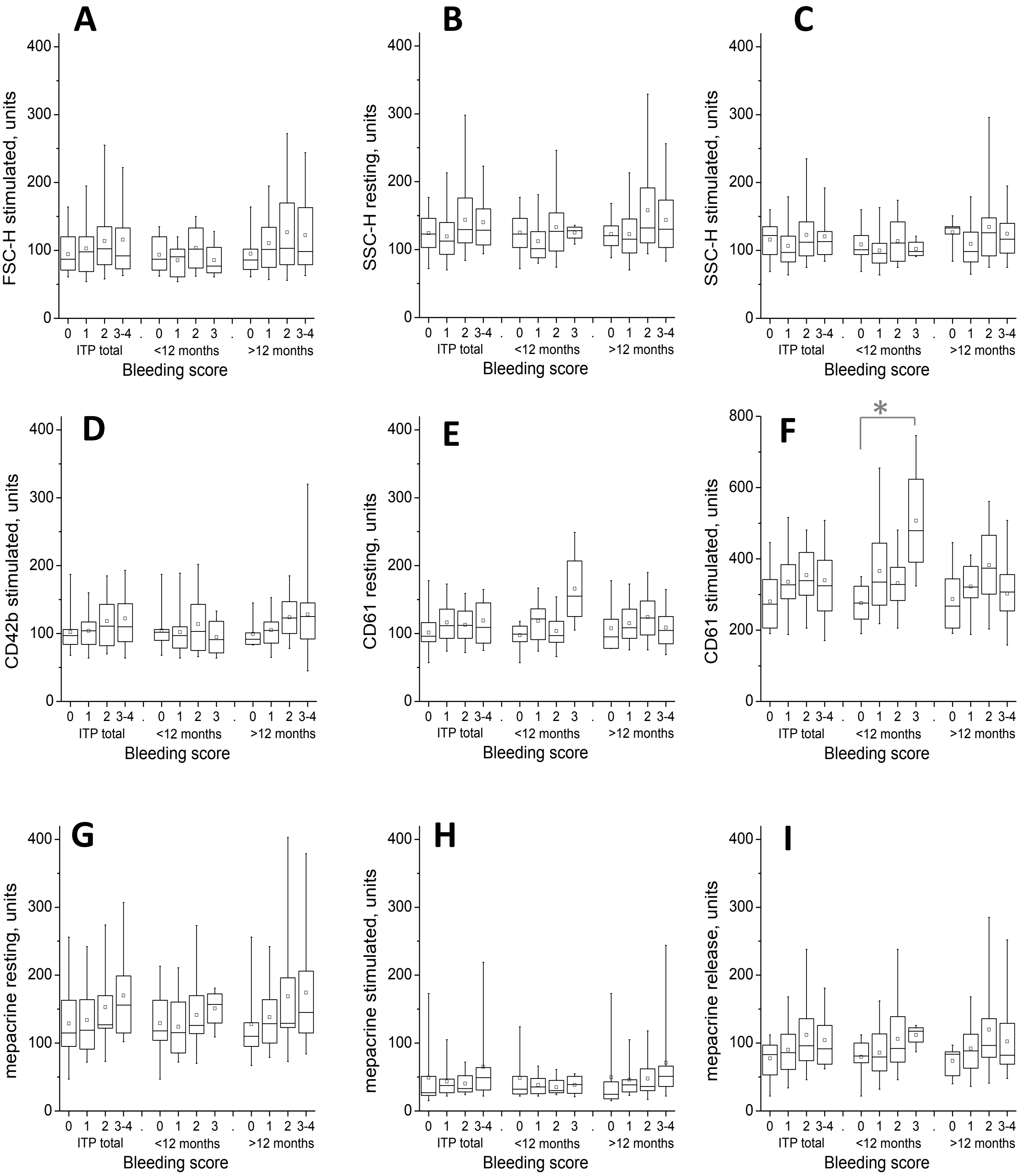

Figure S6. Platelet function and bleeding degree in non-treated ITP. All conditions and notation are as in Fig. S4.

Supplement Figure S7

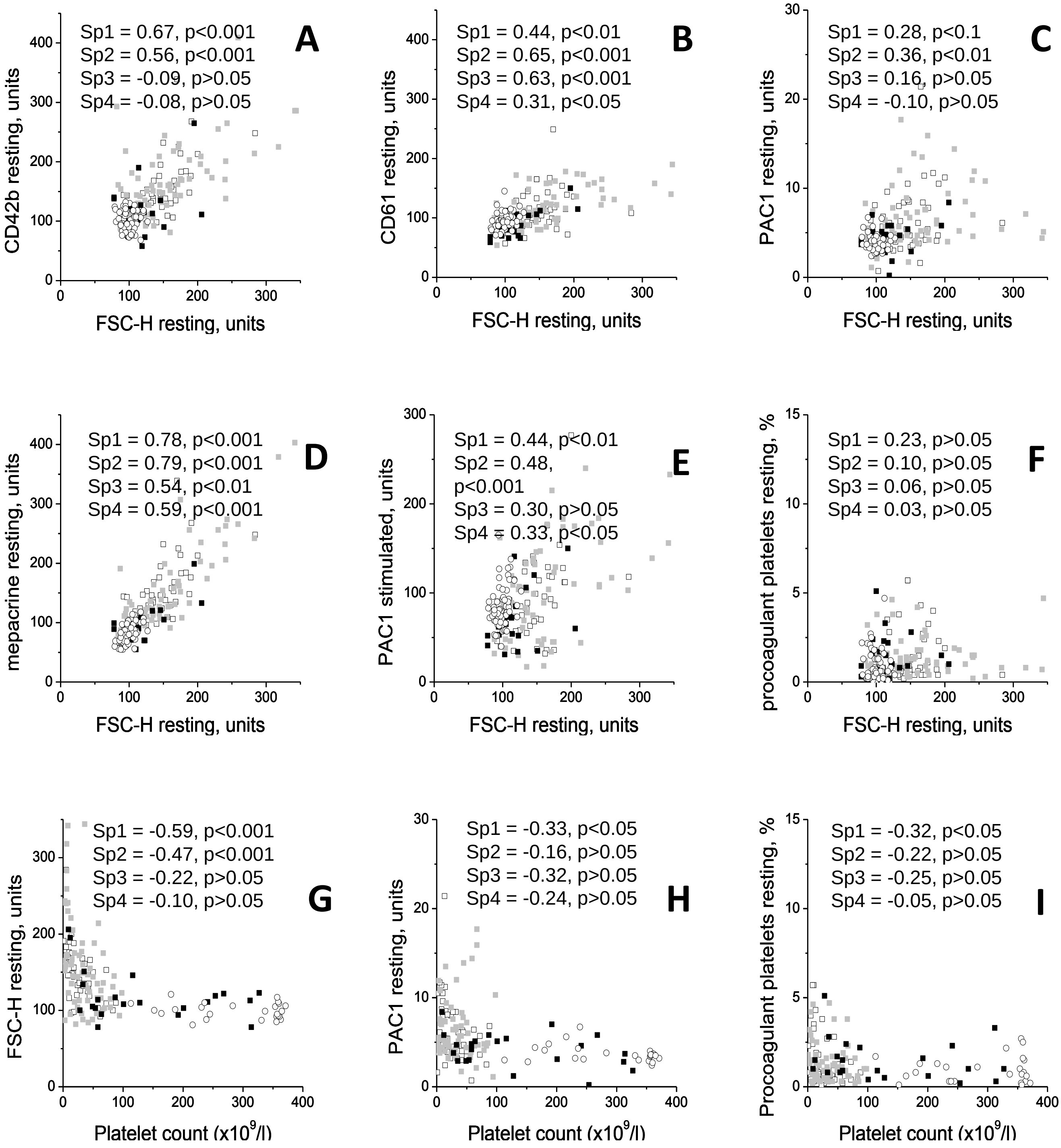

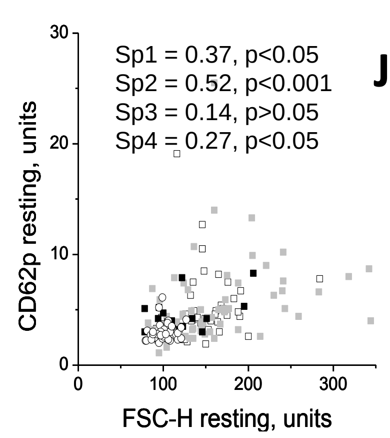

1- □ acute and persistent ITP
2- ■ chronic ITP
3- ■ ITP on romiplostim
4- ○ healthy children

Figure S7. Correlation of the parameters. Correlation plots for the sensitive parameters of platelet function.

Supplement Figure S8

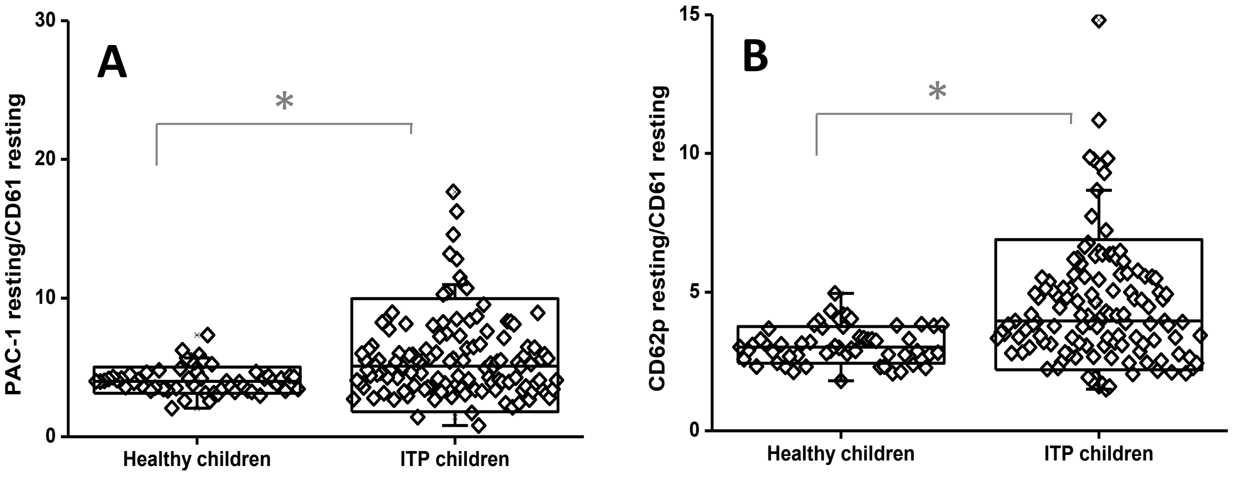

*Figure S8. A. PAC1 MFI in resting platelets normalized to the parameter proportionate to platelet size (CD61 MFI) in resting state for healthy children (n=57, left) and children with ITP (n=151, right). B. CD62p MFI in resting platelets normalized to CD61 MFI in resting platelets for healthy children (left) and children with ITP (right). * p<0,001.*

Supplement Figure S9

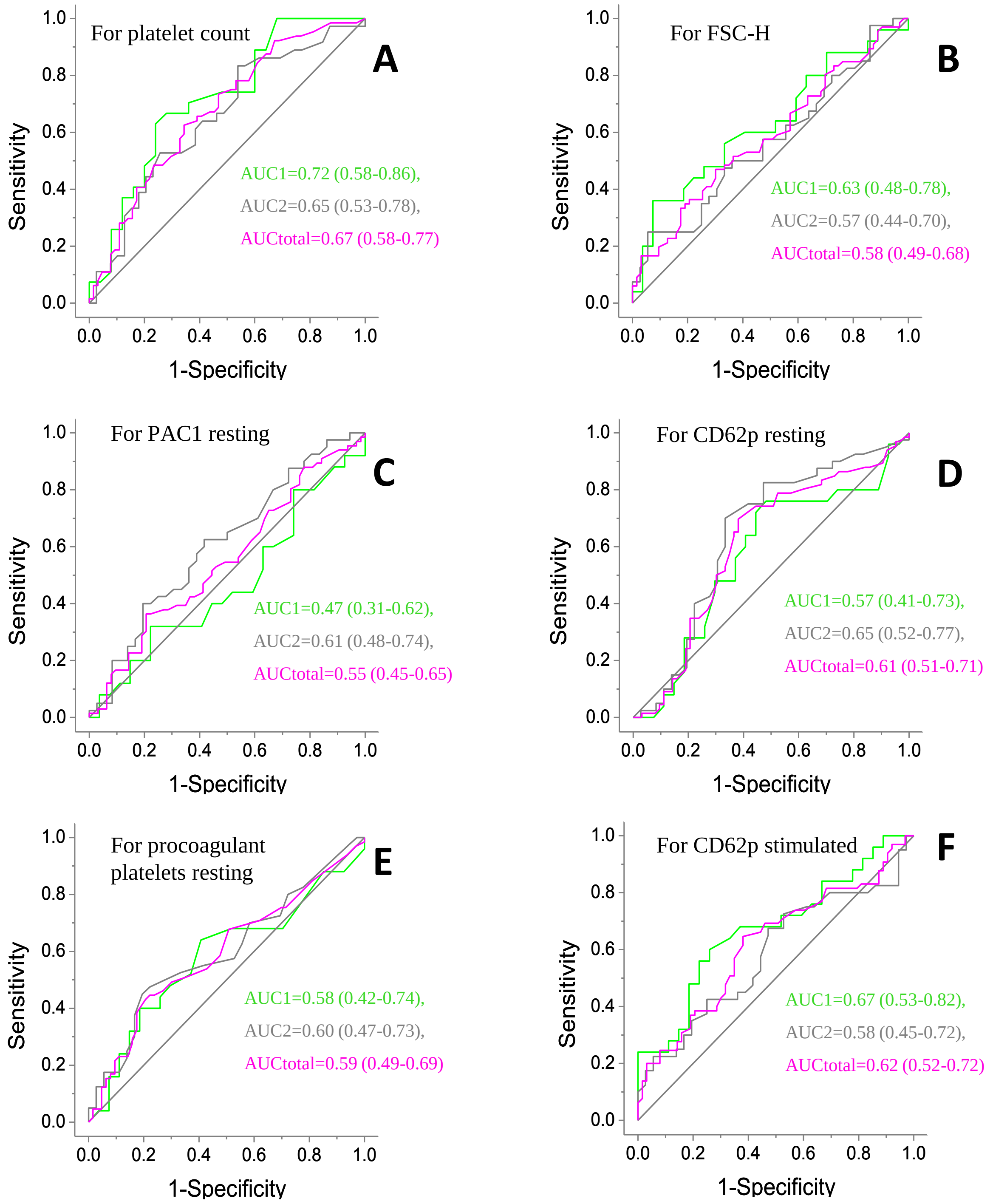

Figure S9. ROC curves of risk factors to predict bleeding in acute+persistent ITP (1, light green), chronic ITP (2, grey) and total ITP patients (pink). Sensitivity and specificity for bleeding complications.

Supplement Figure S10

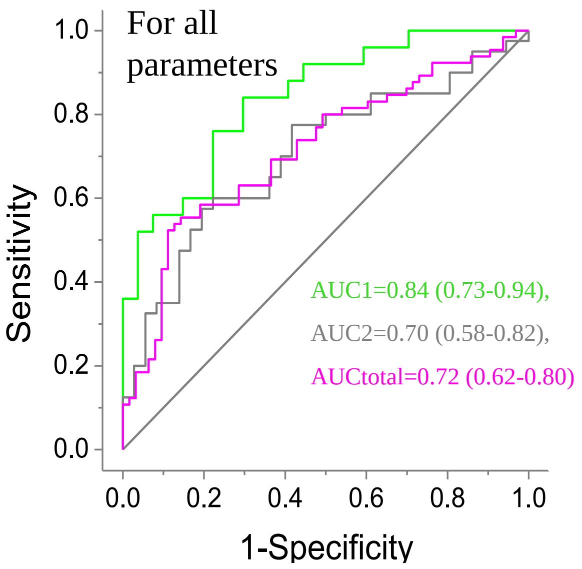

Figure S10. Multivariate analysis of risk parameters (including platelet count, FSC-H resting, PAC1 resting, CD62p resting and stimulated, procoagulant platelets resting) to predict bleeding in ITP. Sensitivity and specificity for bleeding complications.

Supplement Figure S11

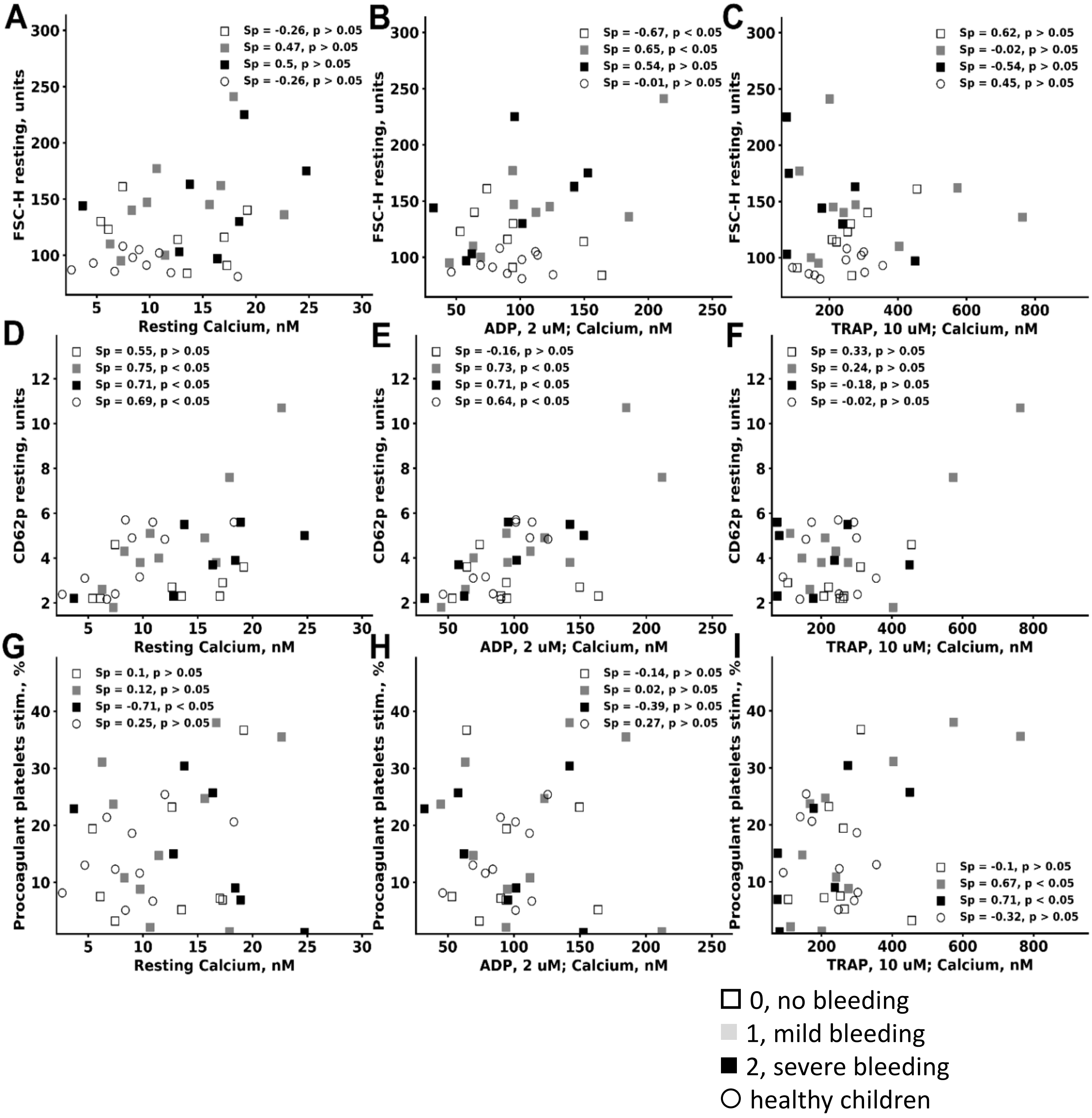

Figure S11. Correlation of the flow cytometry parameters. Correlation plots for the parameters of platelet function and platelet signaling among ITP patients with different bleeding severity. (A-C) No correlation between cytosolic calcium in resting (A), 2 µM ADP activated (except for ITP patients with mild bleeding, B) or 10 µM TRAP-6 activated (C) platelets and platelet FSC-H was observed. (D-E) Cytosolic calcium in resting (D) and 2 µM ADP activated (E) activated platelets correlated with CD62p binding by resting platelets (except for ITP patients with no bleeding). (F) No correlation was observed between cytosolic calcium in 10 µM TRAP-6 stimulated platelets and CD62p binding by resting platelets. (G,H) No correlation between cytosolic calcium in resting (G) and 2 µM ADP activated (H) and procoagulant platelets amount upon stimulation was observed. (I) Cytosolic calcium in 10 µM TRAP-6 stimulated platelets correlated with procoagulant platelets amount in mild and severe bleeding patients with ITP.

Supplement figure S12

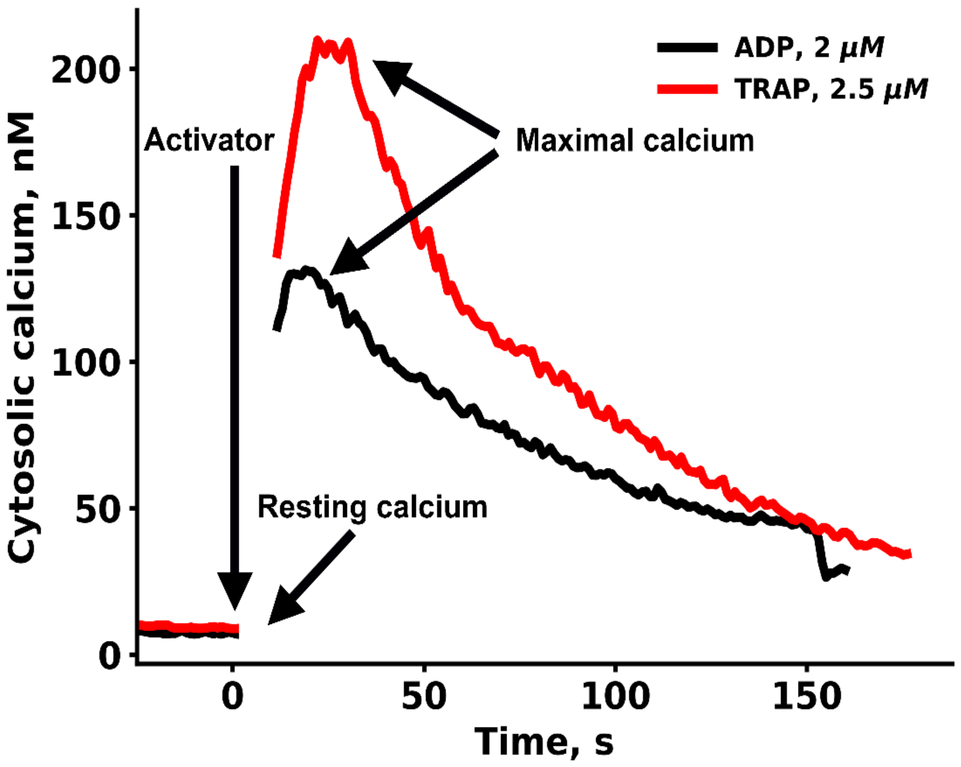

Figure S12. Typical calcium curves, obtained during platelet signaling assessment.
