# Supplementary material for: Platelet function and bleeding at different phases of childhood immune thrombocytopenia
Source: Sci Rep. 2021 Apr 30;11:9401. doi: 10.1038/s41598-021-88900-6 (PMC8087794; doi:10.1038/s41598-021-88900-6)
Supplement: Supplementary file 2 — Supplementary Tables. [file 41598_2021_88900_MOESM2_ESM.docx]

**Platelet function and bleeding at different phases of childhood immune thrombocytopenia**

Anastasia A. Ignatova^1,2,3^*, Elena V. Suntsova ^1^*, Alexey V. Pshonkin ^1^, Alexey A. Martyanov^1,3,4,5^, Evgeniya A. Ponomarenko^1,6^, Dmitry M. Polokhov^1^, Daria V. Fedorova^1^, Kirill A. Voronin^1^, Natalia N. Kotskaya^1^, Natalia M. Trubina^1^, Marina V. Krasilnikova^1^, Selima Sh. Uzueva^1^, Irina V. Serkova^1^, Galina S. Ovsyannikova^1^, Ksenia I. Romanova^1^, Lili A. Hachatryan^1^, Irina I. Kalinina^1^, Viktor E. Matveev^1^, Maya N. Korsantiya^1^, Natalia S. Smetanina^1^, Dmitry A. Evseev^1^, Maria N. Sadovskaya^1^, Kristina S. Antonova^1^, Anna L. Khoreva^1^, Pavel A. Zharkov^1^, Anna Shcherbina^1^, Anastasia N. Sveshnikova^1,3,4,7^, Aleksey A. Maschan^1^, Galina A. Novichkova^1^, Mikhail A. Panteleev^1,3,4,8^

^1^ National Medical Research Center of Pediatric Hematology Oncology and Immunology named after Dmitry Rogachev, Moscow, Russia

^2^ Shemyakin-Ovchinnikov Institute of Bioorganic Chemistry of the Russian Academy of Sciences, Moscow, Russia

^3^ Center for Theoretical Problems of Physicochemical Pharmacology of the Russian Academy of Sciences, Moscow, Russia

^4^ Faculty of Physics Lomonosov Moscow State University, Moscow, Russia

^5^ Institute for Biochemical Physics (IBCP), Russian Academy of Sciences (RAS), Moscow, Russia

^6^ Faculty of Biology Lomonosov Moscow State University, Moscow, Russia

^7^ Department of Normal Physiology Sechenov First Moscow State Medical University, Moscow, Russia

^8^ Faculty of Biological and Medical Physics Moscow Institute of Physics and Technology, Dolgoprudny, Russia

* Equal contribution

Table S1. Patients with non-ITP thrombocytopenia

| **Diagnosis** | **Number** | **Gender** | **Age, median (range) , years** | **Platelet count** |
| --- | --- | --- | --- | --- |
| Leukemia (AML and JMML) | 5 | 2m, 3f | 2.5 (0.8-16) | 51 (15-84) |
| Aplastic anemia | 5 | 2m, 3f | 11.0 (3-17) | 62 (5-65) |
| MYH-9-associated thrombocytopenia | 4 | 4m, 0f | 8.0 (2-15) | 24 (3-58) |
| Wiskott-Aldrich syndrome | 7 | 7m, 0f | 5.0 (1-12) | 38 (11-114) |

Table S2. Logistic regression of risk factors for bleeding in patients with ITP

| Variable | Acute+persistent ITP | Chronic ITP | Total ITP patients |
| --- | --- | --- | --- |
|  | OR (95% CI), p | | |
| Platelet count | 0.972  (0.948-0.997), p=0.03 | 0.981  (0.963-0.999), p=0.04 | 0.978  (0.964-0.992), p=0.003 |
| PAC1, resting | 0.953  (0.807-1.125), p=0.57 | 1.090  (0.960-1.238), p=0.19 | 1.042  (0.951-1.141), p=0.38 |
| CD62p, resting | 0.977  (0.816-1.170), p=0.80 | 1.026  (0.898-1.172), p=0.71 | 1.009  (0.909-1.121), p=0.86 |
| Procoagulant platelets, resting | 1.239  (0.837-1.836), p=0.28 | 1.517  (0.922-2.496), p=0.10 | 1.340  (0.984-1.826), p=0.06 |
| FSC-H, resting | 1.015  (0.997-1.034), p=0.11 | 1.006  (0.998-1.015), p=0.14 | 1.008  (1.000-1.016), p=0.04 |
| CD62p, stimulated | 1.025  (1.002-1.048), p=0.03 | 1.009  (0.998-1.021), p=0.11 | 1.013  (1.003-1.024), p=0.01 |

Table S3. Logistic regression with multiple variables for bleeding risk in patients with ITP

| № | Variables | Acute+persistent ITP | Chronic ITP | Total ITP patients |
| --- | --- | --- | --- | --- |
|  |  | OR (95% CI), p | | |
| 1 | Platelet count | 0.950 (0.911-0.992),  p=0.02 | 0.984 (0.962-1.007),  p=0.18 | 0.979 (0.961-0.997),  p=0.02 |
|  | +PAC1, resting | 0.615 (0.404-0.938),  p=0.02 | 1.066 (0.929-1.222),  p=0.36 | 0.984 (0.887-1.104),  p=0.75 |
|  | +CD62p, resting | 0.824 (0.594-1.143),  p=0.25 | 0.909 (0.771-1.071),  p=0.26 | 0.872 (0.757-1.004),  p=0.05 |
|  | +Procoagulant platelets, resting | 2.173 (1.107-4.262), p=0.02 | 1.569 (0.771-1.071),  p=0.13 | 1.424 (0.976-2.076),  p=0.07 |
|  | +FSC-H, resting | 0.995 (0.967-1.025),  p=0.76 | 1.002 (0.989-1.015),  p=0.78 | 1.001 (0.990-1.013),  p=0.85 |
|  | +CD62p, stimulated | 1.031 (0.995-1.068),  p=0.10 | 1.009 (0.998-1.021),  p=0.11 | 1.009 (0.9946-1.023),  p=0.23 |
| 2 | Platelet count | 0.957 (0.926-0.989),  p=0.01 |  |  |
|  | +PAC1, resting | 0.682 (0.488-0.952),  p=0.02 |  |  |
|  | +Procoagulant platelets, resting | 1.843 (0.991-3.426), p=0.05 |  |  |
| 3 | Platelet count |  |  | 0.974 (0.958-0.990),  p=0.002 |
|  | +CD62p, resting |  |  | 0.887 (0.778-1.011),  p=0.07 |
|  | +Procoagulant platelets, resting |  |  | 1.381 (0.975-1.956), p=0.07 |
